# HIV/STD prevalence and test uptake among african in Guangzhou, China: an analysis of data from hospital-based surveillance

**DOI:** 10.1186/s12879-023-08590-5

**Published:** 2023-09-13

**Authors:** Mingzhou Xiong, Menglan Yang, Peizhen Zhao, Shujie Huang, Cheng Wang

**Affiliations:** 1 Dermatology Hospital of Southern Medical University, Guangzhou, Guangdong China; 2 Guangdong Center for Skin Diseases and Sexually Transmitted Infection Control, Guangzhou, Guangdong China; 3 Southern Medical University Institute for Global Health, Guangzhou, Guangdong China; 4 ZheJiang Provincial People’s Hospital BiJie Hospital, Bijie, China

**Keywords:** HIV, STD, African migrant, Cross-sectional study

## Abstract

Human immunodeficiency virus (HIV) and sexually transmitted diseases (STDs) cause substantial morbidity and mortality both in African and China. However, there is limited data available on the prevalence of HIV/STDs and the uptake of testing experience ever during in China among African migrants. A venue-based survey was conducted at a tertiary hospital in Guangzhou to investigate the prevalence of HIV/STDs through laboratory testing and identify the associated factors. A total of 200 eligible participants completed the survey and bring into the analysis from April to October 2019, and the temporary visitors were excluded. The prevalence rates of HIV, syphilis, NG, CT, and HBsAg among the participants were 1.0%, 2.5%, 1.0%, 1.5%, and 5.5%, respectively. The overall reported rate of HIV/STD testing was 37.0%, with rates of 23.0% for HIV, 16.5% for syphilis, 12.5% for NG, 6.5% for genital herpes, 5.0% for condyloma acuminata, and 2.5% for CT. HIV/STD testing was associated with living environment in Guangzhou, having medical insurance in China, and utilizing health services in China in the past year. HIV/STDs are prevalent among Africans in Guangzhou, and the epidemic is likely to spread due to a significant proportion of unprotected sexual behaviors and low rates of HIV/STD testing. Urgent interventions, including targeted health education, promotion of health service utilization, and active surveillance of HIV/STDs, are needed to reduce the risk of HIV/STD transmission.

## Introduction

Africans in China refer to individuals who are citizens of African countries, identify themselves as African or of African origin, and either reside in China [1]. The number of African in China increased rapidly due to the closer economic cooperation and enhanced educational opportunities [2]. Prior to the onset of the COVID-19 epidemic, an estimated 400,000 to 500,000 Africans lived in China within a twelve-month period [3]. The recent liberalization of China’s entry and exit policy is expected to result in a surge of foreign arrivals [4].

Migration is recognized as a significant, large-scale social change that impacts the spread of human immunodeficiency virus (HIV) and sexually transmitted diseases (STDs), posing challenges to the effectiveness of national Acquired Immune Deficiency Syndrome (AIDS)/ Human Immunodeficiency Virus (HIV) intervention strategies [5]. Studies have identified factors such as partner separation, limited knowledge about HIV risk, and reluctance to undergo HIV testing as contributing factors to an increased risk of HIV infection among Africans in America [6]. Researchers from London have also found a correlation between low contraceptive use among female migrants and an increased risk of STD infections [7]. In the context of migrants, Africans in China predominantly consist of sexually active young adults, indicating a higher risk of disease transmission within the African community and between Africans and the resident population [8]. Furthermore, the low utilization of health services, including HIV/STD testing services, among African migrants makes it challenging to gather accurate and detailed data on HIV/STD infections in this group [9]. Both health-related behaviors and unknown infection statuses pose significant threats to the well-being of Africans in China. Therefore, the growing number of African migrants necessitates further improvements to China’s policies and measures concerning HIV/STD prevention.

Previous research has primarily focused on the health status and healthcare utilization issues among Africans in China, revealing a higher prevalence of chronic diseases such as diabetes, cardiovascular diseases, and cerebrovascular diseases compared to the resident population [10]. It is worth noting that African migrants, due to living in crowded communities with poor sanitation, also face an elevated risk of communicable diseases, including influenza and tuberculosis [11]. Moreover, this group demonstrates a lower willingness to seek medical services when faced with health problems and shows reluctance in seeking s even after engaging in high-risk sexual behaviors [12]. While some studies have reported self-reported HIV/STD prevalence among Africans in China [10], there is a lack of confirmatory laboratory test results. This study aims to explore the prevalence of HIV/STDs through laboratory testing and identify factors associated with HIV/STD test behavior among Africans in Guangzhou.

## Methods

### Study design and participants

This study was conducted in Guangzhou, which is home to a significant population of Africans. According to official statistics at the end of 2019, there were 13,652 Africans residing in Guangzhou, and even during the COVID-19 epidemic, a substantial African population remained [13]. The research took place from April to October 2019 and utilized a venue-based survey conducted at a hospital well-known among African migrants in Guangzhou, particularly in the Sanyuanli, Xiaobei, and Taojin areas, which are predominantly inhabited by Africans. Eligible participants who visited the hospital and provided consent were invited to take part in the study. The inclusion criteria for participants were as follows: being a national of an African country, having spent at least one month in total in Guangzhou, being at least 18 years old, and willing to provide informed consent. Participants who were unable to communicate in Chinese, English, or French, those who were unable to provide blood or urine sample, and those with diagnosed mental disorders were excluded from the study.

### Data collection

A comprehensive and anonymous questionnaire was developed to gather information on demographic characteristics and health-related behaviors. The questionnaire was designed based on a review of relevant literature and fieldwork experiences, and was further refined by experts in African migrant research. To facilitate participants who were proficient in English, French, or Chinese, the questionnaire was available in all three languages. Trained investigators with extensive experience in serving African migrants conducted the survey in the dedicated consulting room, ensuring privacy and confidentiality. Prior to administering the questionnaire, participants provided written informed consent. Then, the participants were completed the questionnaire by themselves, and they can ask investigators for help if they do not understand the content of the questionnaire. Following the questionnaire survey, each participant was provided with a complimentary test package, which included screenings for HIV, Syphilis, Neisseria gonorrhoeae (NG), Chlamydia trachomatis (CT), and Hepatitis B surface Antigen (HBsAg). Urine sample was sent to the Dermatology Hospital of Southern Medical University for testing NG and CT infections, while blood sample was tested for HIV, Syphilis, and HBsAg at the study site (Xinshi Hospital). The test results were promptly communicated to the participants. For those who tested positive for Syphilis, NG, CT, or HBsAg, the study hospital offered normative diagnosis and treatment services. Participants who received a preliminary positive result for HIV screening would be asked to provide another blood sample and for confirmatory testing in Guangzhou Center for disease control (CDC). We would follow up the test procedures and recommend subsequent linkage to care services for participants with positive result in the confirmatory test, and participants with negative results in confirmatory test would be excluded for HIV infection.

### Test method for STD/HIV

Blood sample was collected to test for HIV, Syphilis, and HBsAg. For the initial screening of HIV antibodies, a commercially available latex method was employed using the Human Immunodeficiency Virus 1/2 Antibody Test Kit (China). Syphilis testing was carried out using *the Treponema pallidum* particle agglutination test (SERODIA®-TP*PA, Japan) and the Syphilis Toluidine Red Untreated Serum Test (TRUST, China). A positive result was confirmed when both TPPA and TRUST tests yielded positive results. Urine specimen was utilized for testing CT and NG using the Cobas4800 system (Roche Molecular Systems, Branchburg, NJ, USA). Antibodies to HBsAg were detected using an enzyme-linked immunosorbent assay (ELISA) with the Diagnostic Kit for Hepatitis B Virus Surface Antigen from China.

### Outcome measures

The primary outcome of this study was to determine the prevalence of HIV/STD among participants. This was measured as the proportion of participants who tested positive for HIV, Syphilis, NG, CT, and HBsAg based on the results of blood and urine tests conducted during the research. The secondary outcome focused on the HIV/STD test rate, which was defined as the proportion of participants who had ever undergone any of the HIV/STD tests available in China. This included tests for HIV, Syphilis, NG, Genital herpes, Condyloma acuminate, and CT, as reported in the questionnaire survey.

Demographic information collected included gender, age, marital status, educational level, annual income, religious belief, travel purpose, and living place. Health-related behaviors, such as sexual behavior, number of sex partners, condom use, medical insurance coverage, utilization of health services, history of chronic diseases, and infectious diseases, were also recorded.

### Statistical analysis

The information collected from the questionnaire survey was entered into Epidata 3.1 using a dual input format. Subsequently, the data was exported from Epidata 3.1 and merged with the results obtained from the blood and urine tests. All data analysis was performed using IBM SPSS 24.0 (IBM, NY, USA). The demographic characteristics of the participants were presented and categorized based on their history of HIV/STD testing in China. Logistic regression modeling was conducted to examine the associations between demographic characteristics, health-related behaviors, and HIV/STD test rates. Multivariable logistic regression models were utilized to adjust for potential confounding variables, including gender, age and marital status. The results of the logistic regression analysis were reported as crude odds ratios (OR) and adjusted odds ratios (aOR) with corresponding 95% confidence intervals (CI). Variables that demonstrated statistical significance in the logistic regression model were presented. A p-value of less than 0.05 was considered statistically significant.

## Results

### Demographic characteristics of the participants

During the study period, 214 eligible participants visited the study site, while nine of them rejected the survey, three participants did not complete the questionnaires, and two participants did not provide blood and urine samples, resulting in a final sample size of 200 participants included in the analysis. The study included a majority of male participants (65.5%), with an average age of 36.1 ± 9.3 years. The majority of participants belonged to the young adult age group of 18–49 years (90%). In terms of marital status, 53.0% of participants were married. In regard to education, 34.0% had completed high school, while 35.5% had a university education. In terms of religious affiliation, the majority identified as Christians (69.5%). Regarding the purpose of their stay in China, 74.5% reported coming for business purposes. The participants primarily resided in hotels (54.0%) (Table 1).

**Table 1:** Demographics characters of participants

	Total (n, %)	HIV/STD test		p-value
		Yes (n, %)	No (n, %)	
**Gender**				0.885
Male	131(65.5)	48(64.9)	83(65.9)	
Female	69(34.5)	26(35.1)	42(34.1)	
**Age**				0.157
18–29	59(29.5)	26(35.1)	33(26.2)	
30–39	73(36.5)	29(39.2)	44(34.9)	
40–49	48(24.0)	14(18.9)	34(27.0)	
50–59	17(8.5)	3(4.1)	14(11.1)	
60 and above	3(1.5)	2(2.7)	1(0.8)	
**Marital Status**				**0.011**
Married	106(53.0)	29(39.2)	77(61.1)	
Unmarried	85(42.5)	41(55.4)	44(34.9)	
Divorced (Widowed)	9(4.5)	4(5.4)	5(4.0)	
**Education**				0.187
Below high school	49(24.5)	15(20.3)	34(27.0)	
High school	68(34.0)	21(28.4)	47(37.3)	
College/university	71(35.5)	32(43.2)	39(31.0)	
Above university	12(6.0)	6(8.1)	6(4.8)	
**Annual Income**				0.080
Below 13,000RMB	47(23.5)	24(34.3)	23(19.0)	
13,000RMB -	49(24.5)	17(24.3)	32(26.4)	
32,500RMB-	50(25.0)	13(18.6)	37(30.6)	
65,000RMB-	45(22.5)	16(22.9)	29(24.0)	
**Religious Belief**				0.624
Christianity	139(69.5)	54(73.0)	85(67.5)	
Islamism	43(21.5)	15(20.3)	28(22.2)	
None	18(9.0)	5(6.8)	13(10.3)	
**Travel Purpose**				0.132
Business	149(74.5)	48(64.9)	101(80.2)	
Study	31(15.5)	17(23.0)	14(11.1)	
Other	20(10)	9(12.1)	11(8.7)	
**Living Place in Guangzhou**				0.109
Hotel	108(54.0)	32(43.2)	76(60.3)	
Renting house	67(33.5)	29(39.2)	38(30.2)	
Other*	25(12.5)	13(17.6)	12(9.6)	

*: Including self-purchased house, dormitory, and no fixed residence

### HIV/STD infection prevalence

Based on the laboratory test results, it was found that 2 participants (1.0%) were infected with HIV, 5 participants (2.5%) with Syphilis, 2 participants (1.0%) with NG, 3 participants (1.5%) with CT, and 11 participants (5.5%) with HBsAg. Among them, only one participant had a co-infection of CT and HBsAg. Regarding the history of HIV/STD testing in China, 74 participants (37.0%) reported having undergone any testing for HIV/STD. The test rates for HIV, Syphilis, NG, CT, Genital Herpes, and Condyloma Acuminata were 23.0%, 16.5%, 12.5%, 6.5%, 5.0%, and 2.5%, respectively (Table 2).

**Table 2:** HIV/STD prevalence and test rate among African mi﻿grants

Variables	Frequency (n)	Percentage (%)
**HIV/STD Prevalence**	22	11.0
HIV	2	1.0
Syphilis	5	2.5
NG	2	1.0
CT	3	1.5
HBsAg	11	5.5
**HIV/STD test rate**	74	37.0
HIV	46	23.0
Syphilis	33	16.5
NG	25	12.5
CT	13	6.5
Herpes progenitalis	10	5.0
Condyloma acuminata	5	2.5

### Health-related behaviors

47% of the participants reported engaging in sexual behaviors while in Guangzhou, with the majority of their sexual partners being African (78.7%). Among these participants, 31.0% reported never using condoms with their regular partners, while only 28.7% reported consistent condom use. Regarding temporary sexual partners, 36.2% reported using condoms all the time, while 27.5% reported never using condoms. Only 19.5% of the participants reported having health insurance in China. Furthermore, 52.0% utilized health services in Guangzhou in the past year. The most commonly reported chronic diseases among the participants were high blood pressure (5.0%) and diabetes (5.0%). Additionally, 23 (11.5%) participants had been infected with Influenza and 16 (8.0%) had contracted typhoid fever in the past year (Table 3).

**Table 3:** Health-related behaviors

	Total (n, %)	HIV/STD test		p-value
		Yes (n, %)	No (n, %)	
**Sexual Behaviors in Guangzhou**				**0.001**
No	106(53.0)	28(37.8)	78(61.9)	
Yes	94(47.0)	46(62.2)	48(38.1)	
**Sexual partners in Guangzhou**				0.378
African partner	74(78.7)	36(75.0)	38(82.6)	
Chinese partner	11(11.7)	5(10.4)	6(13.0)	
Other	5(5.3)	4(8.3)	1(2.2)	
Missing	4(4.3)	3(6.3)	1(2.2)	
**Condom Use with Regular Sex Partners**				0.738
Every time	25(27.5)	12(48.0)	13(28.3)	
Most of the time	14(15.4)	7(50.0)	7(15.2)	
Some time	18(19.8)	11(61.1)	7(15.2)	
Never	27(29.7)	11(40.7)	16(34.8)	
No Regular Sex Partners	7(7.7)	4(8.9)	3(6.5)	
**Condom Use with Temporary Sex Partners**				0.786
Every time	25(36.2)	14(56.0)	11(28.2)	
Most of the time	16(16.2)	8(50.0)	8(20.5)	
Some time	9(13.0)	6(66.7)	3(7.7)	
Never	19(27.5)	8(42.1)	11(28.2)	
No Temporary Sex Partners	12(7.1)	6(14.3)	6(15.4)	
**Medical Insurance**				**< 0.001**
No	161(80.5)	49(66.2)	112(88.9)	
Yes	39(19.5)	25(33.8)	14(11.1)	
**Health Service in Guangzhou in the Past Year**				**0.013**
No	96(48.0)	27(36.5)	69(54.8)	
Yes	104(52.0)	47(63.5)	57(45.2)	
**Diagnosed chronic diseases**				
Hypertension	10(5.0)	4(40.0)	6(60.0)	0.530
Diabetes	10(5.0)	4(40.0)	6(60.0)	0.530
Cardiovascular disease	4(2.0)	1(25.0)	3(75.0)	0.535
Hyperlipemia	4(2.0)	0	2(100.0)	0.401
Other	6(3.0)	3(50.0)	3(50.0)	0.383
**Infectious diseases in the past year**				
Influenza	23(11.5)	7(30.4)	16(69.6)	0.510
Typhoid fever	16(8.0)	4(25.0)	12(75.0)	0.312
Sexually transmitted disease	5(2.5)	4(80.0)	1(20.0)	0.061
Pulmonary tuberculosis	3(1.5)	3(100.0)	0	0.048
Infectious diarrhea	2(1.0)	0	2(100.0)	0.400

### Factors associated with HIV/STD test rate

After adjusting for gender, age, and marital status, it was observed that participants who resided in hotels had a lower HIV/STD test rate compared to those staying in other locations (including self-purchased house, dormitory, and no fixed residence, adjusted odds ratio [aOR]: 2.57; 95% confidence interval [CI]: 1.06–6.25). On the other hand, participants who had health insurance (aOR: 3.76; 95% CI: 1.66–8.51) demonstrated a higher HIV/STD test rate compared to those without health insurance. Furthermore, participants who had utilized health services in China within the past year (aOR: 2.11; 95% CI: 1.14–3.85) exhibited higher HIV/STD test rates in comparison to those who had not sought health services (Table 4).

**Table 4:** Factors associated with HIV/STD test rate

Variable	STD test rate n(%)	Crude OR (95% CI)	Adjusted OR (95% CI)
Education			
Below high school	15(30.6)	ref	ref
High school	21(30.9)	1.01(0.46, 2.25)	0.94(0.41,2.17)
College/university	32(45.1)	1.86(0.86,4.00)	1.67(0.73,3.81)
Above university	6(50.0)	**2.27(1.02,8.19)**	1.95(0.51,7.44)
**Annual Income**			
Below 13,000RMB	24(51.1)	ref	ref
13,000RMB -	17(34.7)	0.08(0.01,6.31)	0.50(0.22,1.18)
32,500RMB-	13(26.0)	**0.03(0.01,0.94)**	0.44(0.18,1.08)
65,000RMB-	16(35.6)	0.07(0.01,8.95)	0.82(0.33,2.08)
**Living Area in Guangzhou**			
Hotel	32(29.6)	ref	ref
Renting house	29(43.3)	7.79(0.09,653.41)	1.81(0.96,3.42)
Other	13(52.0)	**49.00(1.76,136.34)**	**2.57(1.06,6.25)**
**Condom Use with Regular Sex Partners**			
Every time	12(48.0)	ref	ref
Most of the time	7(50.0)	**0.04(0.01,0.08)**	1.45(0.35,5.94)
Some time	11(61.1)	0.03(0.01,7.69)	2.25(0.58,8.66)
Never	11(40.7)	0.02(0.01,258.72)	1.24(0.30,5.20)
No Regular Sex Partners	4(57.1)	9.31(0.01,197.93)	1.43(0.24,8.62)
**Medical Insurance**			
No	49(30.4)	ref	ref
Yes	25(64.1)	**4.08(1.96,8.52)**	**3.76(1.66,8.51)**
**Health Service utilization in China in the Past Year**			
No	27(28.1)	ref	ref
Yes	47(45.2)	**2.11(1.17,3.80)**	**2.11(1.14,3.89)**

Adjusted with gender, age, and marital status

## Discussion

The prevalence of HIV/STD among African migrants in China remains uncertain. In this study, we established a hospital-based surveillance site and reported the HIV/STD prevalence among African migrants in China based on laboratory test results. We found that the utilization of HIV/STD testing services among African migrants in China is inadequate. Additionally, we identified associations between their HIV/STD testing behaviors and factors such as living place in Guangzhou, medical insurance, and health service utilization in China.

Our study revealed a higher prevalence of HIV (1.0%) and syphilis (2.5%) among African migrants than the physical examination screening data from Guangdong International Travel Health Care (0.03% for HIV, 1.8% for syphilis, respectively), and the HBV prevalence were comparable (5.5% VS 5.8%) [14]. This difference may be attributed to the demographic characteristics of the participants in our study. Specifically, a higher proportion of participants in our study fell within the younger age range (18–49 years: 90.0% vs. approximately 72.3%) and had lower levels of education (high school and below: 58.5% vs. 26.9%). These factors may contribute to a higher proportion of high-risk sexual behaviors [8]. However, when comparing the prevalence of HIV/STD among African migrants with other high-risk groups such as men who have sex with men (HIV: 5.7%, syphilis: 5.8%) and attendees of STD clinics (NG: 11.95%, CT: 12.02%) in China, the prevalence among African migrants still remains relatively low [15–17]. We noticed that co-infection of HIV/STDs, such as HIV and syphilis, CT and NG, were reported frequently in other studies [18, 19], while only one co-infection (CT and HBsAg) has been observed in our study. The limited sample size of our study may lead to this discrepancy, and other potential associated factors were needed to explore in further research. Among the tested HIV/STDs in our research, the prevalence of HBV (5.5%) among African migrants was found to be the highest. However, it was lower than the prevalence in the general Chinese population (6.89%) and the general population of African countries (18.9% in Somalia, 7.8% in Kenya, 8.1% in South Africa, and 8.5% in Uganda) [20–24]. Due to the limited data available in our study, we lack sufficient information to analyze the associated factors of the difference in HBV prevalence between African migrants and the general Chinese and African populations.

Although the HIV/STD prevalence among African migrants in this research was not significantly higher compared to other high-risk groups, it still requires further attention due to the potential risk of HIV/STD infection. The majority of African migrants in this study were young adults, which is consistent with findings from previous studies [25] and indicates a sexually active population. Approximately half of the participants reported engaging in sexual behaviors in Guangzhou, and among them, 31.0% and 27.5% reported never using condoms with regular partners or temporary partners, respectively, highlighting the risk of HIV/STD infection. However, only 23.0% of participants had ever received an HIV test in China, which is considerably lower than the testing rates among African migrants in the United Kingdom (51.8%) and Canada (64.0%) [26, 27], as well as the testing rates reported among individuals aged 15 years and older in South Africa (73.8-76.7%) [28].

The low HIV/STD test rate can be attributed to several factors. Firstly, there was a policy change in China in 2010 that eliminated the requirement for foreigners to provide negative HIV test results as a visa requirement upon entry. Consequently, there are currently no mandatory HIV testing requirements during their stay in China [29]. Additionally, the low medical insurance coverage, which was only 19.5% in this research, is associated with a lower uptake of HIV/STD testing among African migrants. Furthermore, language barriers have been identified as critical factors affecting the HIV/STD testing behavior of African migrants [8], limiting their access to routine medical services and free HIV/STD testing services in China, such as the HIV voluntary counseling and testing program (VCT) [30, 31].

The low testing rate also hinders the offering of timely health care services to treat and prevent HIV. Therefore, it is crucial for the government to actively improve and formulate medical security policies as a priority. Additionally, the following monitoring measures are recommended: (1) Enhance awareness of HIV/STD testing by providing health education in the mother language of African migrants, which should include HIV/STD preventive knowledge and information about nearby health institutions. (2) Increase healthcare utilization rates by improving healthcare availability, including the provision of targeted medical services at African migrant residences and enhancing the language proficiency of healthcare providers. (3) Establish a persistent and active HIV/STD surveillance network among international migrants in China to gather health information that is currently unavailable through the passive surveillance system.

### Limitations

Several limitations of this study should be acknowledged. Firstly, although the study site covered the three major African migrant settlements, namely Sanyuanli, Xiaobei, and Taojin, the limited sample size may introduce sampling bias, making it challenging to accurately reflect the level of HIV/STD infection in this population. Secondly, some potential key factors associated with HIV/STD testing among African migrants in China, such as the HIV/STD test experiences in their home country and sexual orientation, were neglected in this survey. Therefore, further research is needed to collect more comprehensive information and provide a deeper understanding of the topic. Lastly, as with any questionnaire survey, recall bias may have existed for some participants, which is inevitable.

## Conclusion

HIV/STD prevalence is a significant concern among Africans in China, and the epidemic is likely to continue spreading due to the high prevalence of unprotected sexual behaviors and low rates of HIV/STD testing. Interventions are urgently required to address this issue, including targeted health education, promotion of healthcare utilization, and consistent active surveillance of HIV/STDs. These measures are necessary to reduce the risk of HIV/STD transmission.

## Data Availability

All data relevant to the study was included in the article.
